# Managing Patent Foramen Ovale (PFO) in Adults With Cryptogenic Stroke and Lymphoma: A Multidisciplinary Perspective

**DOI:** 10.7759/cureus.40316

**Published:** 2023-06-12

**Authors:** Noman Khalid, Muhammad Abdullah, Sacide S Ozgur, Sherif Elkattawy, Fayez Shamoon

**Affiliations:** 1 Internal Medicine, St. Joseph's Regional Medical Center, Paterson, USA; 2 Public Health & Community Medicine, Shaikh Khalifa Bin Zayed Al-Nahyan Medical and Dental College, Shaikh Zayed Federal Postgraduate Medical Institute, Shaikh Zayed Medical Complex, Lahore, PAK; 3 Cardiology, St. Joseph's Regional Medical Center, Paterson, USA

**Keywords:** lymphoma, bubble study, paradoxical emboli, cryptogenic stroke, patent foramen ovale

## Abstract

​​Cryptogenic strokes are strokes with no clear underlying cause. Patent foramen ovale (PFO) is believed to be one of the causes of cryptogenic strokes. To manage such cases, closing the PFO is usually considered an option. We report a case of a middle-aged male with lymphoma who presented with an altered mental status due to a stroke, which, on investigation, was found to be due to an underlying PFO. This report explores the factors that must be considered when making the decision to close the PFO and emphasizes the vital role of a multi-disciplinary team in determining the best course of action for patients with cryptogenic strokes.

## Introduction

Patent foramen ovale (PFO) is a connection between the right and left atria of the heart that is present in embryonic life and typically closes in infancy [1]. In most infants, the atrial opening closes early in life; however, a PFO persists in 25% of healthy adults and mostly remains asymptomatic [2]. A PFO can provide a pathological channel for the thrombus to embolize into the left-sided circulation in patients with deep venous thrombosis (DVT) or other venous thromboembolic disorders. Techniques to investigate and confirm PFO include transthoracic echocardiography (TTE), transesophageal echocardiography (TEE), and transcranial Doppler (TCD) [2]. These investigations, with the injection of normal saline contrast (Bubble study), can help detect right to left shunts associated with PFO. The mainstay of the medical treatment includes systemic anticoagulation and antiplatelet therapy [3]. The transcatheter approach is considered the most effective method for PFO closure [4]. Asymptomatic PFO patients are not surgically or medically treated [3].

## Case presentation

A 54-year-old male with a history of hypertension, smoking, cocaine abuse, traumatic splenectomy, and CD 30+ peripheral T-cell lymphoma (on brentuximab vedotin, adriamycin, bleomycin sulfate, vinblastine sulfate, and Dacarbazine) presented to the emergency department (ED) for further evaluation of altered mental status. He had fallen and hit his head at home, leading to unresponsiveness and involuntary jerking movements of his limbs. Upon admission, the patient's vital signs were blood pressure of 159/92 mmHg, pulse of 92 beats/min, respiratory rate of 16 breaths/min, and oxygen saturation (SpO2) of 88% on room air. On physical examination, he was drowsy but arousable, not oriented to time, place, or person, and had decreased strength and jerking movements of the right upper extremity. However, four hours later, the patient had returned to normal orientation and had 5/5 strength throughout, with normal muscle tone and bulk. He also had intact pinprick and light touch sensations. The patient's head was normocephalic and non-traumatic, with grade-I bilateral peripheral edema and decreased breath sounds on both lung bases.

**Figure 1:**
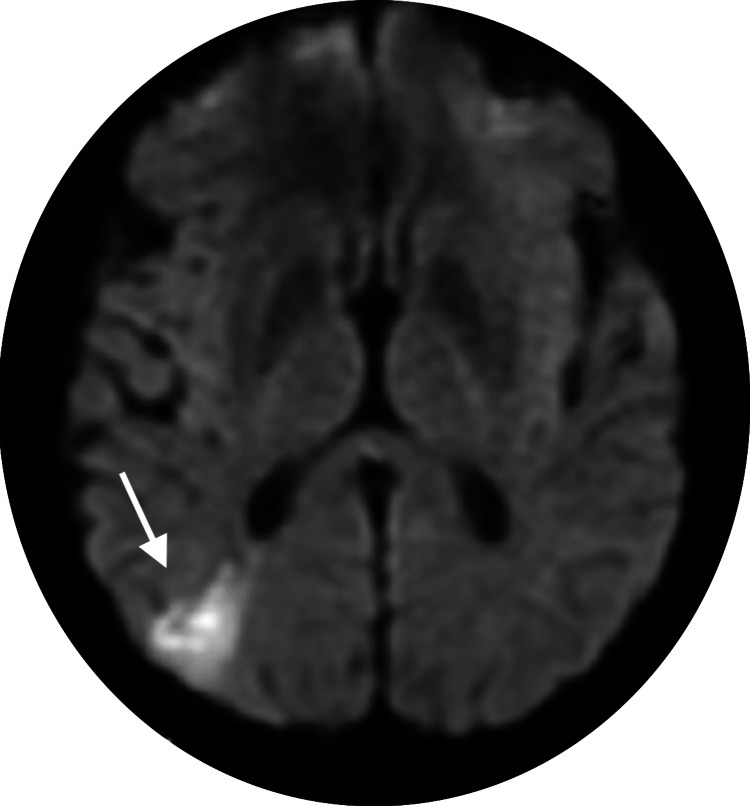
Focal wedge-shaped area of diffusion restriction in right occipital lobe indicating acute infarction on axial DWI DWI: diffusion-weighted imaging

The patient had hypoxemia on arterial blood gas analysis (ABG) and elevated troponin-I levels, with a 4706 ng/L peak value. D-dimer was 13.38 mcg/ml, and procalcitonin was 0.08 ng/mL. EKG showed sinus rhythm, submillimeter ST elevation in inferior leads, and submillimeter ST depression in aVL, which warranted coronary angiography. Venous duplex ultrasound revealed lower extremity left-sided acute deep vein thrombosis (DVT). A CT head without contrast was negative for intracranial hemorrhage, large territorial infarct, or other acute intracranial abnormalities but positive for old/chronic right cerebellar infarct. MRI brain with and without contrast showed acute infarct in the posterior circulation (Figure 1) distribution and old infarct and encephalomalacia in the right cerebellum. EEG showed no epileptiform discharges or lateralizing signs. Two-dimensional (2D) Echo showed a left ventricular ejection fraction of 55-60%, mitral valve regurgitation, mild septal paradoxical motion, inferobasal, and inferolateral basal mild hypokinesis. Transesophageal echocardiography revealed a positive bubble study and a Grade 1 PFO (1 to 5 bubbles) with a size of less than 2 mm (Video 1). Cardiac catheterization showed mild right coronary artery disease with 20% stenosis.

**Video 1:** Trans-esophageal echocardiography agitated saline study (Bubble study) showing Grade 1* PFO * Grade 1 PFO (1 to 5 bubbles) PFO: patent foramen ovale

At admission, the patient's National Institutes of Health Stroke Scale (NIHSS) score was 1 for the level of consciousness, and neurology was consulted, which deemed the patient was not a tissue plasminogen activator (tPA) candidate. ST-elevation myocardial infarction (STEMI) code was also called due to EKG changes, and cardiology was consulted. The patient was started on high-intensity statins, aspirin, clopidogrel, and heparin infusion and later transitioned to apixaban for planned PFO repair after discharge. Dual antiplatelet therapy was discontinued as the patient began systemic anticoagulation for DVT management. Prophylactic antibiotics were also started on admission but later discontinued due to negative blood culture. The patient was switched from heparin to apixaban before discharge. The patient was discharged after a few days of stability with a plan for outpatient follow-up.

After discharge, the patient was followed up for PFO closure; the patient’s Risk of Paradoxical Embolism (ROPE) score was 5 points; there was a 34% chance that stroke was due to PFO, and a 7% risk for two years recurrence of stroke/TIA. As the patient was already on long-term systemic anticoagulation because of lymphoma and DVT, it was decided not to pursue PFO closure until after the discontinuation of anticoagulation.

## Discussion

This study is a case of an adult with acute ischemic stroke, acute encephalopathy, left peroneal DVT, cocaine use disorder, and acute hypoxic respiratory failure; upon work-up, he was found to have a PFO. The patient was evaluated by cardiology, neurology, critical care, infectious disease, and oncology consults. Thus, this patient is an example of a multidisciplinary team treating a patient with PFO and other disorders, making shared decisions for better healthcare outcomes.

The patient underwent cardiac catheterization by the cardiology team to rule out any underlying disease because of the EKG changes and other coronary artery disease risk factors.

The diagnosis of PFO is routinely made by using a bubble test using transthoracic echocardiography (TTE) or transesophageal echocardiography (TEE) [1]. The PFO can also be directly visualized by transesophageal echocardiography (TEE), the gold standard for diagnosis [1]. In our patient, the PFO was confirmed by a TEE with a bubble study.

In our patient, the cause of the paradoxical thromboembolism may be lymphoma, chemotherapy, cocaine abuse, or alcohol intoxication, which are well-known causes of thrombophilia.

For patients with PFO, primary prevention of stroke by a PFO closure is not recommended [1]. However, in PFO patients with a history of stroke or transient ischemic attack, the closure of the PFO may need to be considered [5]. Recurrent strokes in PFO patients often require antiplatelet or anticoagulant therapy, and PFO closure is considered only after identifying specific factors [5,6].

The decision to consider PFO closure as a secondary preventive measure for stroke in adults should be guided by high-risk anatomic features such as atrial septal aneurysm and large shunt size [6,7]. The effectiveness of PFO closure, in combination with antiplatelet therapy, for secondary stroke prevention remains uncertain for patients without these high-risk features [6]. A meta-analysis of randomized trials suggests that transcatheter closure may effectively prevent recurrent strokes in certain patients with recent cryptogenic strokes, with an increased risk of atrial fibrillation [8]. Other complications related to closure may include atrial fibrillation, pneumothorax, hemothorax, cardiac tamponade, and aortic dissection [6]. However, PFO closure by a transcatheter device with antiplatelet therapy after a recent cryptogenic stroke has been recommended by some experts over antiplatelet therapy alone [9]. In our patients, no high-risk anatomical features would necessitate PFO closure.

Medical therapy for the prevention of recurrent stroke after cryptogenic stroke, in the absence of DVT, includes aspirin, and in patients with DVT, anticoagulants are recommended [3]. When not going for PFO closure, anticoagulation is preferred over antiplatelet therapy, as used in our case [9]. In our case, initially, PFO closure was planned for our patient. However, input from all relevant specialties led us to conclude that PFO closure was unnecessary for our patient due to the absence of high-risk anatomical features of the PFO and the recommendation to continue indefinite anticoagulation due to the high risk of recurrent DVT.

## Conclusions

In conclusion, when considering PFO closure as a management option for stroke, it is essential to conduct a comprehensive evaluation to exclude other potential causes. This evaluation should involve discussions among specialists, such as neurologists and cardiologists, and consider the input of primary care, hematology, or rheumatology specialists, as needed. This collaborative approach becomes particularly important in cases with ambiguous diagnostic results or unique medical histories.

Shared decision-making plays a vital role in the process, as it allows for a thorough examination of the limited potential benefits of PFO closure in preventing recurrent strokes while also considering the associated procedural risks. By carefully weighing these factors, healthcare providers can make informed decisions that prioritize their patients' well-being and long-term health outcomes.
